# B cells promote granulomatous inflammation during chronic *Mycobacterium tuberculosis* infection in mice

**DOI:** 10.1371/journal.ppat.1011187

**Published:** 2023-03-08

**Authors:** Yong Chen, Sushma Bharrhan, Jiayong Xu, Tarina Sharma, Yanhua Wang, Padmini Salgame, Jinghang Zhang, Kievershen Nargan, Adrie J. C. Steyn, Paul J. Maglione, John Chan

**Affiliations:** 1 Department of Medicine (Infectious Diseases), New Jersey Medical School, Newark, New Jersey, United States of America; 2 Department of Pathology, Albert Einstein College of Medicine, Bronx, New York, United States of America; 3 Department of Microbiology & Immunology, Albert Einstein College of Medicine, Bronx, New York, United States of America; 4 Africa Health Research Institute, University of KwaZulu Natal, Durban, KwaZulu-Natal, South Africa; 5 Department of Microbiology, Centers for AIDS Research and Free Radical Biology, University of Alabama at Birmingham, Birmingham, Alabama, United States of America; 6 Department of Medicine, Boston University School of Medicine, Boston, Massachusetts, United States of America; Portland VA Medical Center, Oregon Health and Science University, UNITED STATES

## Abstract

The current study reveals that in chronic TB, the B cell-deficient μMT strain, relative to wild-type (WT) C57BL/6 mice, displays in the lungs lower levels of inflammation that are associated with decreased CD4^+^ T cell proliferation, diminished Th1 response, and enhanced levels of interleukin (IL)-10. The latter result raises the possibility that B cells may restrict lung expression of IL-10 in chronic TB. These observations are recapitulated in WT mice depleted for B cells using anti-CD20 antibodies. IL-10 receptor (IL-10R) blockade reverses the phenotypes of decreased inflammation and attenuated CD4^+^ T cell responses in B cell-depleted mice. Together, these results suggest that in chronic murine TB, B cells, by virtue of their capacity to restrict expression of the anti-inflammatory and immunosuppressive IL-10 in the lungs, promote the development of a robust protective Th1 response, thereby optimizing anti-TB immunity. This vigorous Th1 immunity and restricted IL-10 expression may, however, allow the development of inflammation to a level that can be detrimental to the host. Indeed, decreased lung inflammation observed in chronically infected B cell-deficient mice, which exhibit augmented lung IL-10 levels, is associated with a survival advantage relative to WT animals. Collectively, the results reveal that in chronic murine TB, B cells play a role in modulating the protective Th1 immunity and the anti-inflammatory IL-10 response, which results in augmentation of lung inflammation that can be host-detrimental. Intriguingly, in tuberculous human lungs, conspicuous B cell aggregates are present in close proximity to tissue-damaging lesions manifesting necrosis and cavitation, suggesting the possibility that in human TB, B cells may contribute to the development of exacerbated pathology that is known to promote transmission. Since transmission is a major hindrance to TB control, investigating into whether B cells can shape the development of severe pulmonic pathological responses in tuberculous individuals is warranted.

## Author summary

*Mycobacterium tuberculosis* remains a major public health threat worldwide. Balancing of the protective response and potentially tissue-damaging inflammation during tuberculous infection influences disease outcome. Chronic TB can be associated with lung-damaging inflammation, resulting in necrotization and cavity formation, pathological processes that are conducive to the transmission of *M*. *tuberculosis*. Restricting transmission is an important measure for TB control. We initiated experiments to characterize the role of B cells in modulating lung inflammation in chronic TB, the phase of infection during which tissue-damaging inflammation is most likely to occur. The results suggest that B cells can drive the development of lung inflammation in chronic TB by enhancing the levels of protective interferon (IFN)-γ-producing CD4^+^ T cells while restricting the expression of the immunosuppressive, anti-inflammatory IL-10. Thus, in chronic infection, B cells may contribute to optimal TB control but paradoxically promote lung pathology. Understanding how B cells regulate lung inflammation in TB may help the design of strategies that can reduce transmission, thereby achieving better TB control.

## Introduction

*M*. *tuberculosis* remains a significant public health burden globally [1]. It has been estimated that there were 10 million new cases of tuberculosis (TB) in 2019, and 1.4 million people succumbed to the infection [1]. As the hallmark pathological process of TB, the granulomatous inflammation, a highly complex and heterogeneous immune reaction, influences both mycobacterial containment and immune-mediated inflammatory damage during *M*. *tuberculosis* infection [2–8]. This paradox of the tuberculous granuloma reflects a complex spectrum of host immunity against the tubercle bacillus, ranging from inadequate to excessive responses, with productive yet pathologically benign containment of *M*. *tuberculosis* as the preferred moderation of these two extremes [2–8]. It is thus likely that the host response to *M*. *tuberculosis* involves regulatory mechanisms that intricately balance adequate immunity with minimal inflammatory pathologic damage. Indeed, in approximately 90% of individuals, persistent *M*. *tuberculosis* infection is contained locally in the lungs without apparent clinical symptoms or severe pathology [9]. When TB reactivation of the latently infected loci occurs, immunopathologic damage can result from an excessive host response that is not adequately protective [10].

In chronic TB, tissue-damaging inflammation can lead to the development of immunopathology manifesting as necrosis and/or cavitation [11–13]. These pathological processes play a significant role in promoting the transmission of *M*. *tuberculosis*, thereby perpetuating tuberculous infection [12, 14]. Components of immunity that determine the progression of immunopathologic inflammation, particularly in chronic persistent *M*. *tuberculosis* infection, remain incompletely defined; and it is generally thought that an excessive inflammatory response to the tuberculous bacillus could dampen TB control and concomitantly damage lung tissues [2–8]. To further complicate the treatment of TB patients, the tissue-damaging pathological process can result in residual compromised pulmonary functions even after curative treatment of the infection [15]. Thus, understanding the mechanisms that regulate the development of inflammation in a tuberculous host with chronic infection could help design therapies for better TB control by mitigating transmission and for preventing undesirable lung damage caused by *M*. *tuberculosis*-induced immunopathology.

The multifunctional attributes of B cells enable this lymphocyte subset to significantly modulate immune reactions in a wide variety of pathophysiological states [16–18], including inflammation associated with infection [19–22]. B cells form prominent aggregates in tuberculous lung tissues of multiple species such as mice, non-human primates, and humans [23–25]. Evidence exists that these B cells aggregates are a component of ectopic germinal centers that have been shown to regulate local immune responses [26,27], including those in tuberculous lungs ([28–31] & reviewed in [23]). Accumulating evidence suggest that B cells and humoral immunity play a significant role in influencing *M*. *tuberculosis* infection and disease outcomes [23,32]. The role of B cells in regulating the levels of inflammation in tuberculous tissues, particularly during the chronic phase of infection, however, has been understudied. Nevertheless, results derived from a murine TB model involving the μMT B cell-deficient mouse strain suggest a pro-inflammatory role for B cells in the chronic phase of infection [33]. But that study only conducted histological examination of hematoxylin-eosin (H&E)-stained tissue sections from the chronically infected mice [33]. Given the significance of the inflammatory response in chronic TB in the context of tissue damage and transmission, we initiated experiments to characterize the effects of B cells on the development of granulomatous inflammation in lungs of mice chronically infected with *M*. *tuberculosis*. Comparing the lung inflammatory response in chronic TB of WT C57BL/6’s with that of their B cell-deficient counterparts [34,35], the present study has provided evidence that in chronic TB, B cells i) can contribute, at least in part, to the development of pulmonic inflammation by enhancing the levels of the protective and pro-inflammatory IFN-γ-producing CD4^+^ T cells in the lungs [9,36]; ii) this Th1 augmentation may be due to the ability of B cells to restrict the expression of the immunoregulatory IL-10 that possesses immunosuppressive and anti-inflammatory properties [37–40]. Thus, B cells can optimize TB control by enhancing Th1 immunity via restriction of IL-10 expression; although this apparently protective B cell/IL-10/Th1 axis may result in the development of lung inflammation that can be detrimental to the host [2–8]. The precise mechanisms by which this pathway modulate lung inflammation warrants further investigation.

## Results

### Diminished pulmonary infiltrates in μMT mice relative to WT C57BL/6’s during chronic infection with *M*. *tuberculosis* Erdman

**Fig 1:**
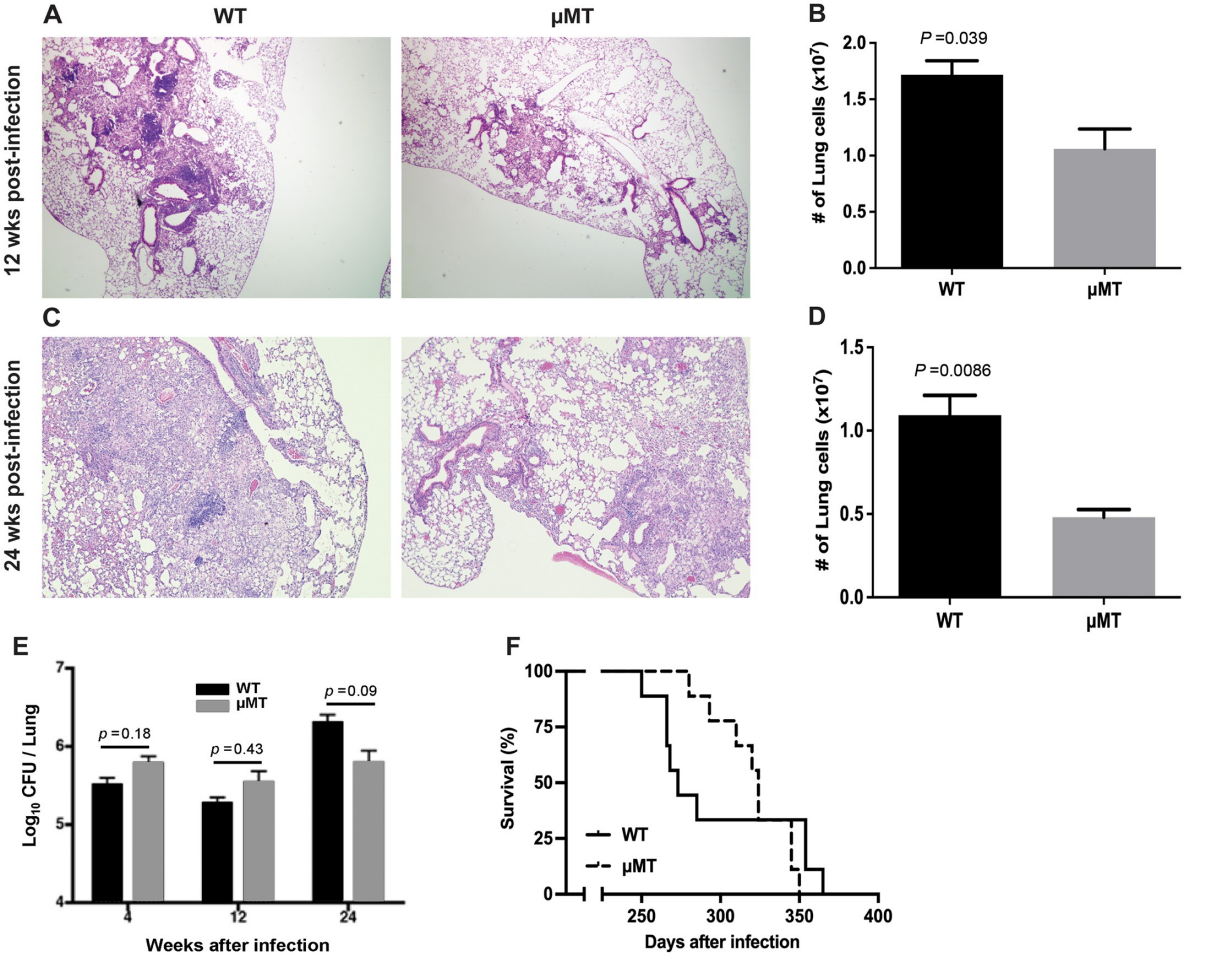
B cell-deficient μMT mice exhibit diminished lung infiltrates relative to WT C56BL/6’s in chronic TB. WT and B cell-deficient μMT mice were aerogenically infected with 100 CFU of *M*. *tuberculosis* Erdman and tissues analyzed by examination of H&E-stained lung sections at 12 weeks (A) and 24 weeks (C) post-infection. Quantification of the number of total lung cells was also carried out at the same time intervals post-inoculation (12 weeks, (B); 24 weeks (D)). These histological studies and enumeration of total lung cell numbers showed that in chronic TB, B cell-deficiency is associated with diminished granulomatous inflammatory response; *n* = 3–4 mice per group per time interval. The results depicted in (A) and (C) are representative of lung sections from 3 mice examined. The data shown in (B) and (D) denote the mean ± SEM (standard error mean). The results shown in (A, B, C, and D) are representative of 2 experiments. (E) Lung mycobacterial burden as measured by culturable bacilli 4, 12, and 24 weeks after a 100 CFU Erdman aerogenic challenge, demonstrating that bacillary loads in μMT and WT mice are comparable throughout the course of infection; *n* = 4 to 5 mice per group. The data depicted denote the mean ± SEM. The results shown are representative of two experiments. (F) B cell-deficient μMT mice exhibited increased median survival relative to WT controls after a 100 CFU aerosol Erdman infection (median survival time: 324 days versus 272 days); *n* = 10 mice per group. The result is demonstrable in three independent experiments.

To begin assessing the role of B cells in modulating lung inflammatory response in mice with chronic TB, we evaluated the level of inflammation by histological analysis of H&E-stained sections of lung tissues procured at 12 weeks and 24 weeks from WT and μMT mouse strains after an aerosol challenge with 100 colony forming units (CFU) of *M*. *tuberculosis* Erdman. The results of these studies revealed that μMT mice exhibit diminished levels of pulmonary granulomatous inflammation relative to WT controls (Fig 1A and 1C). This observation is reinforced by a decrease in total cell numbers in the lungs of B cell-deficient mice compared to WT controls (Fig 1B and 1D). At 12 weeks and 24 weeks post-infection, the total number of lung cells in the μMT mice, compared to the WT C57BL/6’s, decreases by 38% (WT: 1.72 ± 0.13 x 10^7^; μMT: 1.06 ± 0.18 x 10^7^; *p*<0.04) (Fig 1B) and 56% (WT: 1.09 ± 0.12 x 10^7^; μMT: 0.48 ± 0.05 x 10^7^; *p<*0.01) (Fig 1D); respectively. These results are in agreement with that reported previously in a CDC1551 study [33], which has provided evidence that B cells exhibit pro-inflammatory properties during the chronic phase of tuberculous infection. However, the pro-inflammatory role of B cells in the chronic CDC1551 infection study has not been characterized beyond microscopic examination of the histology of H&E-stained tuberculous tissues [33]. Accordingly, we conducted experiments to further characterize how B cells augment the inflammatory response in the lungs of mice with chronic TB.

The diminished granulomatous inflammatory response observed in the μMT mice during the chronic phase of tuberculous infection prompted us to examine whether the absence of B cells alters the ability of a chronically infected host to control *M*. *tuberculosis*. As can be seen in Fig 1E, the lung bacterial loads of the B cell-deficient μMT mice are comparable to that detected in WT C57BL/6’s at 12 weeks and 24 weeks post-infection. This latter observation suggests that the disparate levels of lung granulomatous inflammatory response among the two mouse groups is not due to differences in antigenic load. Rather, the result suggests that B cells play a role, directly or indirectly, in promoting the granulomatous inflammatory in chronic TB. Surprisingly, the B cell-deficient μMT mice display an increased median survival time relative to WT controls (324 days (μMT) versus 272 days (WT)), which is most apparent in the chronic phase of infection (Fig 1F). Although the disparate medium survival time between the two mouse groups falls short of attaining statistical significance, the difference was evident in three independent experiments. Collectively, these results suggest that the decreased inflammatory response in the lungs of B cell-deficient mice does not compromise the capacity of the infected host to control TB for at least up to 6 months post-inoculation (Fig 1E). Rather, the attenuated inflammation is associated with a survival advantage in chronic TB, suggesting that the pro-inflammatory properties of B cells could adversely affect disease outcome in the chronic phase of infection [2–8].

### The presence of B cells promotes cellular expansion and CD4^+^ T cell proliferation in the lung of mice with chronic TB

**Fig 2:**
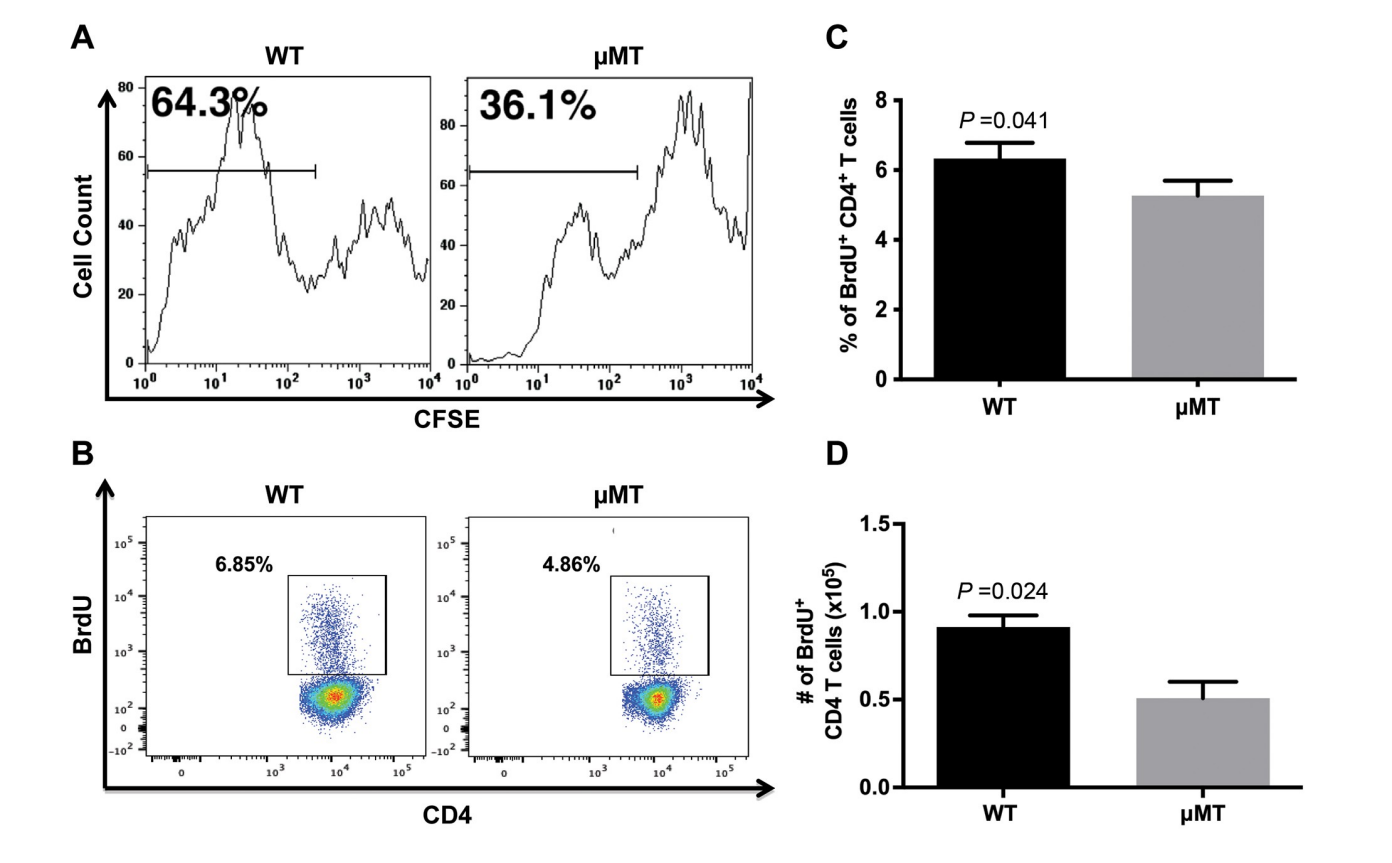
The presence of B cells promotes cellular expansion and CD4^+^ T cell proliferation in the lung of mice with chronic TB. WT and μMT mice were infected with 100 CFU *M*. *tuberculosis* Erdman delivered via aerosolization. Dilution of CFSE by *ex vivo* lung cultures taken 5 months after infection. Cells from 3 mice per group were pooled for the analysis. Data in (B and C) show the frequencies of BrdU^+^ CD4^+^ T cells in the lungs of WT and μMT mice derived from *in vivo* BrdU labeling studies conducted at 5 months post-infection. The data shown in (B) depicts a representative dot plot targeting BrdU^+^ CD4^+^ T cells. Results shown in (C) are mean ± SEM of the frequencies of BrdU^+^ CD4^+^ T cells. Three to 4 mice per group were evaluated. (D) The absolute number of lung BrdU^+^ CD4^+^ T cells at 5 months after infection upon *in vivo* BrdU labeling. The data shown depict mean ± SEM of 3 to 4 mice per group. Data shown are representative of 2 to 3 experiments, except for that in (A), which are derived from 1 study.

B cells can significantly modulate immune responses through a gamut of immunological functions, including the production of cytokines [16–18,20,41] and antibodies [23,32], as well as by serving as potent antigen-presenting cells [23,42–45]. It thus stands to reason that B cells may regulate the local expansion of immune cells in the lungs of tuberculous mice, thereby affecting the levels of lung granulomatous inflammation. Using *ex vivo* CFSE (carboxyfluorescein succinimidyl ester) dilution assay, we observed that at 24 weeks after infection, the levels of expansion of lung cells from the B cell-deficient mice is attenuated relative to that of WT controls. (Fig 2A). This result suggests that the diminished pulmonary infiltrates observed in the μMT mice could, at least in part, be attributed to a reduction in cellular expansion in the lungs. At 24 weeks post-infection, the number of *M*. *tuberculosis* in the lungs of the WT and μMT mice are comparable (Fig 1E), thus ruling out bacterial burden as a factor that leads to the discrepancy in the *ex vivo* cellular proliferation observed among the two groups.

CD4^+^ T cells, which play a significant role in the development of anti-TB immunity [9,46], constitute a major immune cell population in *M*. *tuberculosis-*infected lungs [24]. *M*. *tuberculosis* elicits a robust Th1 response in the lungs of an infected host, and IFN-γ produced by these CD4^+^ T cells possess pro-inflammatory property [36]. B cells, by virtue of their multifunctionality, play an important role in modulating CD4^+^ T cell responses in a range of immunological reactions, including those arising from infection [20,22,47]. We therefore initiated studies to examine whether the CD4^+^ T cell response may be modulated by B cells during chronic tuberculous infection. We began by examining whether CD4^+^ T cells are among the lung cell populations that expand in the *ex vivo* CFSE assay, using *in vivo* bromodeoxyuridine (BrdU) incorporation as a measure of cellular division. The results of this series of experiments revealed that at 24 weeks after infection, the frequency of BrdU^+^ CD4^+^ T cells in the lungs of B cell-deficient mice is lower than that detected in WT controls (Fig 2B and 2C; *p<*0.05), with a decrease of absolute number of lung BrdU^+^ CD4^+^ T cells by 45% (WT: 0.91 ± 0.07 x 10^5^; μMT: 0.5 ± 0.09 x 10^5^; *p*<0.03) (Fig 2D). Thus, the presence of B cells promotes CD4^+^ T cell proliferation in the lungs of mice with chronic TB, which may contribute to the increased pulmonary infiltrates noted in the lungs of WT controls relative to μMT mice.

### B cell-deficient μMT mice, relative to WT C57BL/6 strain, exhibit diminished levels of IFN-γ-producing CD4^+^ T cells during chronic TB

**Fig 3:**
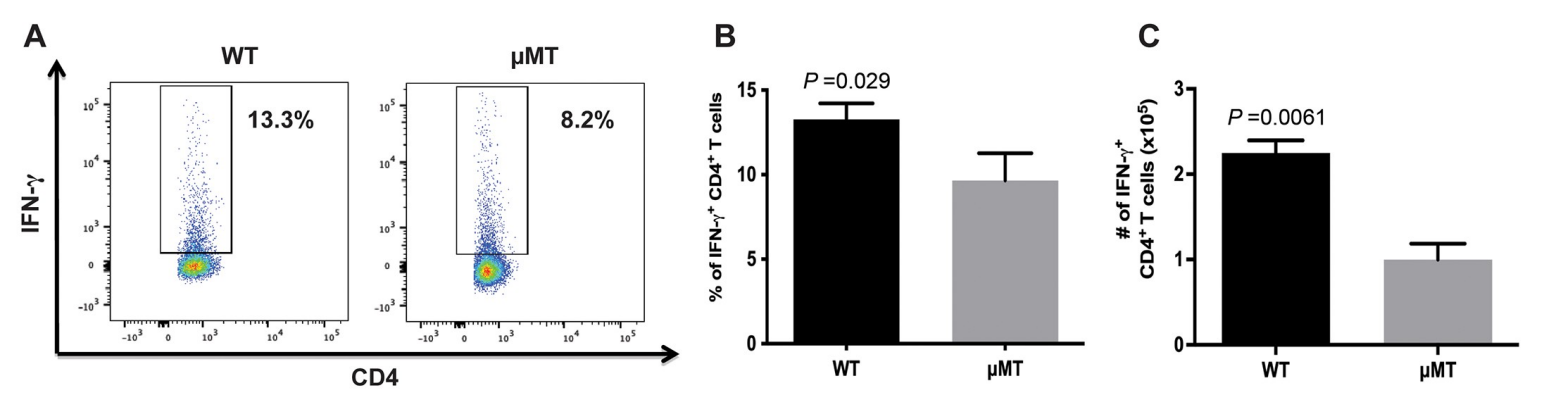
B cell-deficient μMT mice exhibit diminished levels of IFN-γ-producing CD4^+^ T cells during chronic TB. Mice were infected with 100 CFU of *M*. *tuberculosis* Erdman by aerosolization. (A) Representative frequencies of IFN-γ^+^ CD4^+^ T cells detected by flow cytometry at 5 months after infection; *n* = 4 mice per group. (B) Absolute number of lung IFN-γ^+^ CD4^+^ T cells detected 5 months after infection; *n* = 4 mice per group. The results are expressed as mean ± SEM. Data in this figure is representative of 3 similar experiments. The data show revealed the B cell-deficient μMT mice, relative to WT, display an attenuated Th1 response during the chronic phase of tuberculous infection.

As an essential cytokine mediator of anti-TB immunity and a stimulus for inflammation, IFN-γ plays an important role in modulating the outcome of tuberculous infection, disease progression, and the development of granulomatous inflammation [9,36]. In humans, genetic disturbances involving IFN-γ and its regulatory axis are associated with enhanced susceptibility to mycobacteria [48,49]. IFN-γ is essential for the control of acute [50,51] as well as chronic [52] TB in mice. These established roles for IFN-γ in shaping the immune response to *M*. *tuberculosis*, together with the decreased levels of CD4^+^ T cell proliferation observed in B cell-deficient mice (Fig 2B and 2C), have led us to evaluate if B cells influence the frequency of IFN-γ-producing CD4^+^ T cells in the lungs of tuberculous mice during chronic infection. Flow cytometric analysis showed that at 24 weeks post-infection, the frequency of IFN-γ-producing CD4^+^ T cells in the lungs of tuberculous μMT mice is lower than that detected in WT animals (*p<*0.03) (Fig 3A and 3B). Quantification of IFN-γ-producing CD4^+^ T cells revealed that the number of this Th1 population is diminished by 56% compared to that of WT animals (WT: 2.25 x 10^5^ ± 0.15; μMT: 1.0 x 10^5^ ± 0.19; *p*<0.01) (Fig 3C). At this time interval of the chronic infection, the lung bacillary burden is comparable among the two groups of mice (Fig 1E), thus, the discrepancy in the levels of IFN-γ-producing CD4^+^ T cells among the two mouse strains is not due to differences in the numbers of bacteria present in the lungs. Additionally, the comparable bacterial loads between the two mouse groups suggest that the decrease in IFN-γ levels in the lungs of the chronically infected B cell-deficient mice does not compromise their capacity in restricting of *M*. *tuberculosis* growth in the murine TB model used in the present study. Together, these results suggest that B cells can enhance the Th1 response in chronic TB, resulting in increased number of CD4^+^ T cells that produce pro-inflammatory IFN-γ [36] that can promote the development of granulomatous inflammation.

### Increased IL-10 production in the lungs of B cell-deficient μMT mice during chronic infection with *M*. *tuberculosis*

**Fig 4:**
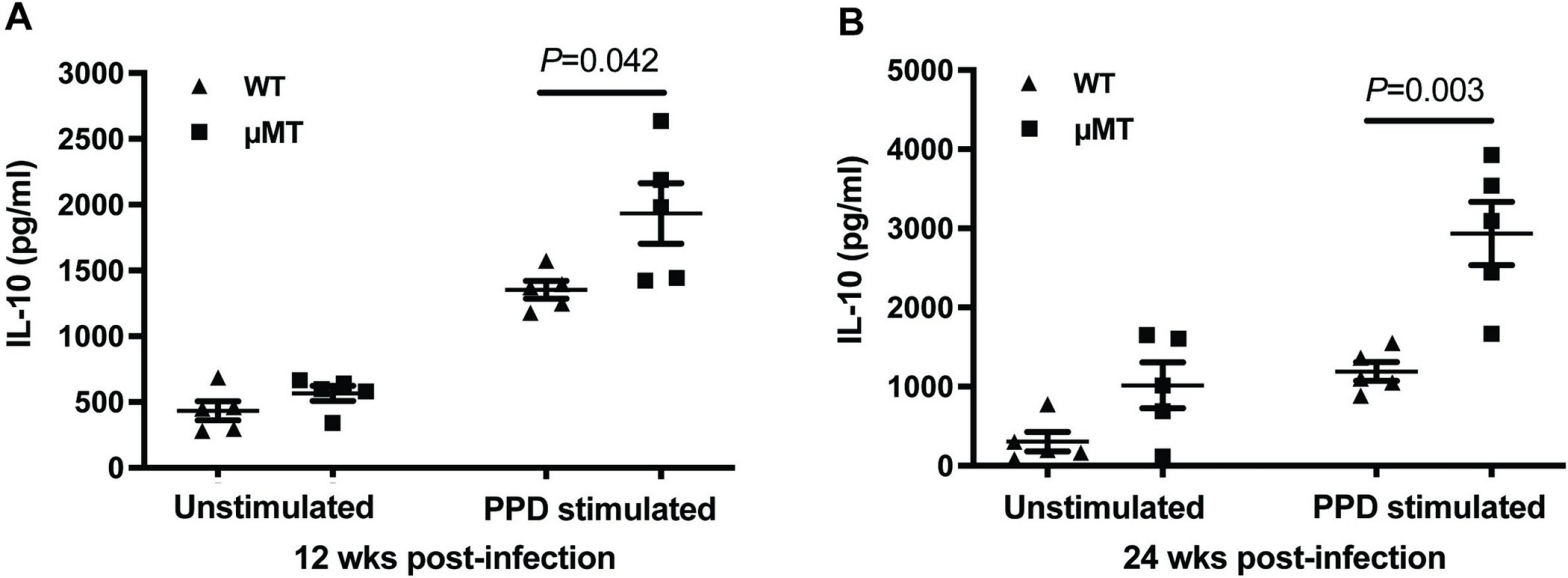
Increased IL-10 production by the lung cells of B cell-deficient μMT mice during chronic infection with *M*. *tuberculosis*. Mice were aerogenically infected with a low dose (100 CFU) of *M*. *tuberculosis* Erdman. *Ex vivo* IL-10 production by lung cells isolated from B cell-deficient μMT and WT control mice was evaluated at 12 weeks (A) and 24 weeks (B) after infection. Single-cell suspension of lung cells were cultured in complete RPMI with or without 10 μg/ml of PPD (Statens Serum Institute, Copenhagen) at 1.0 x 10^7^ cells per ml. After 48 hours of culture, supernatants were collected and subjected to detection of IL-10 as described in “Materials and Methods” section. The results revealed that the levels of expression of lung IL-10 in tuberculous μMT mice are enhanced compared to that observed in WT animals. The data are depicted as mean ± SEM. The results shown in this figure are representative of two independent experiments; *n* = 4 to 5 mice per group per time interval.

A cytokine with anti-inflammatory and immunosuppressive properties [37,38], IL-10 is known to be immunoregulatory during tuberculous infection, and is thought to play a role in balancing protective anti-TB immunity and limiting the development of tissue-damaging inflammation [39,40]. Indeed, ample evidence exists that IL-10 is immunoregulatory in a wide variety of infectious diseases in the development of protective response and inflammation [37,38]. We and others have reported that mice with B cell-deficiency exhibit increased production of IL-10 in the lungs during the acute phase of infection [31,35,53]. The expression of IL-10 in B cell-deficient mice has not been evaluated in the chronic phase of TB. Based on the above, and the diminished levels of granulomatous inflammation detected in the lungs of the chronically infected B cell-deficient mice (Fig 1A–1D), studies were initiated to evaluate the levels of IL-10 in the lungs of WT and μMT mice during the chronic phase of tuberculous infection. The results of these studies demonstrated that at 12 weeks post-infection, the lung cells of B cell-deficient μMT mice generate IL-10, upon PPD stimulation, at a level higher than that of WT animals (an increase of 42.8% compared to WT mice; *p*<0.05) (Fig 4A). This B cell deficiency-associated IL-10 phenotype is also apparent at 24 weeks post-infection, with the PPD-stimulated lung cells of μMT strain producing 150% of the cytokine generated by that of the C57BL/6 mice (*p<*0.005) (Fig 4B). The comparable lung bacterial burden (Fig 1E) among the two mouse groups at the time of analysis: i) excludes the possibility that the observed discrepancy in IL-10 production between the WT and μMT mice is the result of different numbers of *M*. *tuberculosis* in the lungs of infected animals; and ii) suggests that the augmented lung expression of the immunosuppressive IL-10 (Fig 4) in the tuberculous B cell-deficient mice does not compromise TB control in these animals in the chronic phase of infection in the model studied. Together, the data suggest that B cells can directly or indirectly restrict the production of IL-10 in the lungs of an infected host in chronic TB; and that the augmented lung production of the anti-inflammatory IL-10 in the chronically infected μMT mice can contribute to the attenuated granulomatous inflammatory response observed in the B cell-deficient host.

### B cell-depleted WT C57BL/6 mice exhibit diminished granulomatous inflammation and Th1 response and enhanced IL-10 levels in the lungs during chronic *M*. *tuberculosis* infection

**Fig 5:**
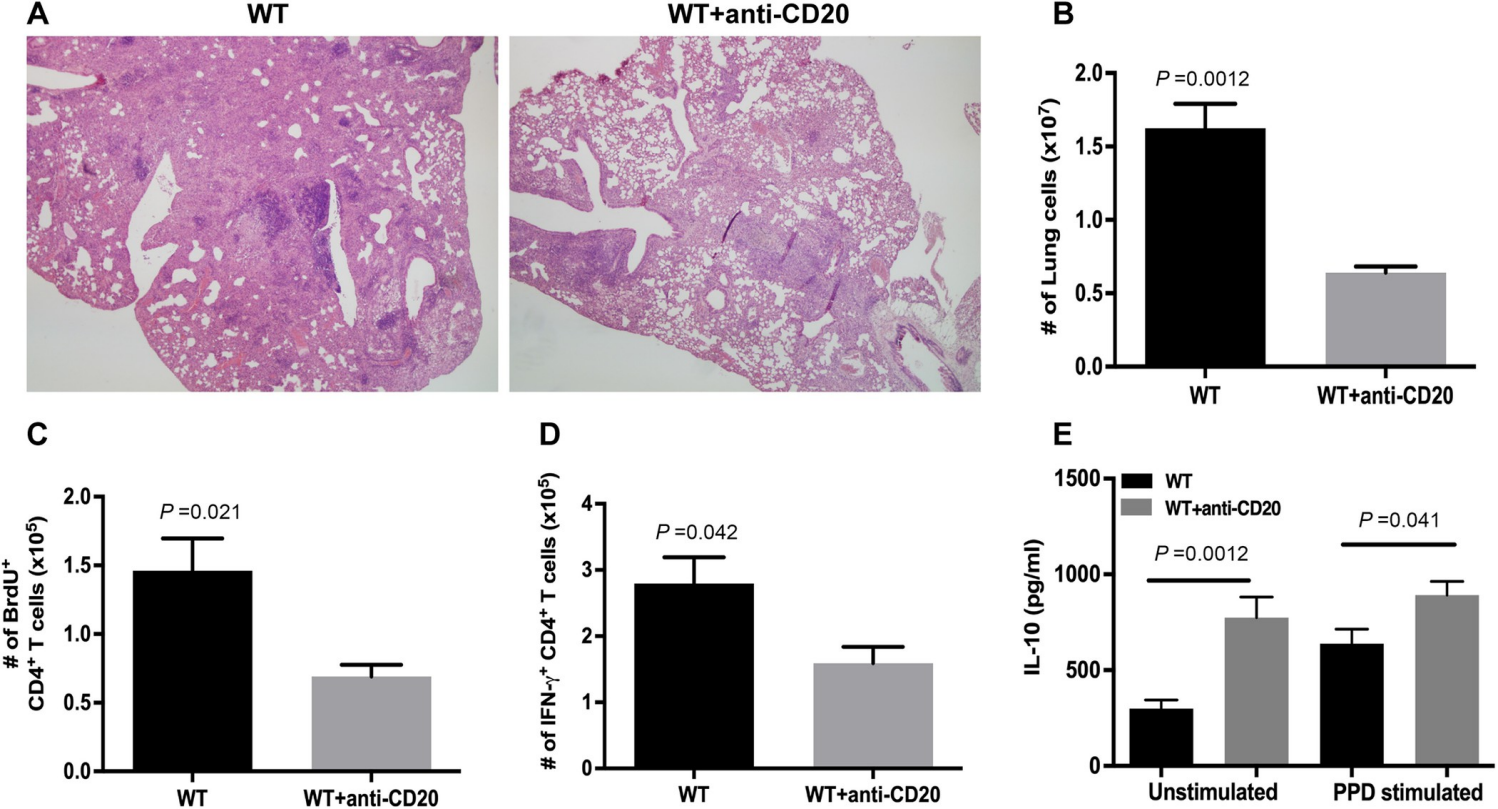
B cell-depleted WT C57BL/6 mice also exhibit diminished granulomatous inflammation and Th1 response and enhanced IL-10 levels in the lungs during chronic *M*. *tuberculosis* infection. Mice were treated with the anti-CD20 mAb 5D2 beginning 2 days prior to a low-dose (100 CFU) aerogenic challenge with *M*. *tuberculosis* Erdman, as described in "Materials and Methods". B cell depletion was maintained throughout the duration of the experiment. The control mouse group (WT) received non-specific rat IgG. The lung tissues were examined at 5 months post-infection. The levels of granulomatous inflammation response were analyzed histologically by light microscopy on H&E-stained lung sections (A) and enumeration of total number of lung cells (B). The level of lung CD4^+^ T cell response was assessed by *in vivo* BrdU labeling to examine the proliferation capacity of this T cell subset (C), as well as by enumeration of IFN-γ-producing CD4^+^ T cells (D). *Ex vivo* evaluation of lung cells for the level of IL-10 production (E) was conducted as described in Fig 4. Four to 5 mice per group were evaluated per group. Data depicted in B, C, D, and E are presented as means ± SEM. The data shown are representative of two experiments. The results demonstrated that the inflammation, Th1 response, and IL-10 phenotypes observed in the μMT mice are recapitulated in mice depleted for B cells.

The above-described results pertaining to the effects of B cells on the lung immune response to *M*. *tuberculosis* in mice with chronic TB derive from the μMT strain rendered B cell-deficient through genetic engineering [34]. To more stringently test the role of B cells in regulating the granulomatous inflammation during chronic TB in mice, B cell-depletion experiments were conducted using the B cell-depleting monoclonal antibody (mAb) 5D2 [35]. As in the μMT model, 5D2-treated, B cell-depleted C57BL/6 mice exhibit an attenuated granulomatous inflammatory response compared to untreated animals during the chronic phase of infection (Fig 5A). The result is supported by the lower number of total lung cells in the B cell-depleted mice (WT: 1.625 ± 0.17 x 10^7^; 5D2-treated: 0.64 ± 0.04 x 10^7^; *p<*0.01) (Fig 5B). As in the μMT model, the decrease in granulomatous inflammation in the B cell-depleted C57BL/6 mice is associated with diminished proliferation of CD4^+^ T cells, as assessed by *in vivo* BrdU labeling study (BrdU^+^ CD4^+^ T cells: WT, 1.46 ± 0.23 x 10^5^; 5D2-treated mice: 0.69 ± 0.08 x 10^5^; *p<*0.03) (Fig 5C) and decreased Th1 response, as assessed by quantification of IFN-γ-expressing CD4^+^ T cells (WT: 2.79 ± 0.39 x 10^5^; 5D2-treated mice: 1.59 ± 0.25 x 10^5^; *p<*0.05 (Fig 5D). As observed in the μMT study, the expression of IL-10 by lung cells of tuberculous B cell-depleted mice is higher than that of control C57BL/6’s not treated with 5D2, with a 160% (*p<*0.01) and 29% (*p<*0.05) increase relative to B cell-sufficient C57BL/6’s in unstimulated and PPD-stimulated cultures; respectively (Fig 5E). Thus, the granulomatous inflammation, CD4^+^ T cell, and IL-10 phenotypes observed in *M*. *tuberculosis-*infected μMT mice are all recapitulated in 5D2-treated B cell-depleted WT C57BL/6’s, strongly suggesting that these manifestations are not simply due to off-target effects resulting from the manipulation of the genome in the generation of the μMT mouse strain. Parenthetically, we had evaluated the frequency and total number of CD4^+^ T cells in parallel in the experiments depicted in Figs 2 and 3 (the μMT study) and Fig 5 (the anti-CD20 study). The results (S1 Fig) show that in the chronic phase of infection, WT mice have a higher number of pulmonic CD4^+^ T cells compared to that detected in the B cell-deficient μMT (S1A Fig) and anti-CD20-treated WT (S1C Fig) mice. By contrast, the CD4^+^ T cell frequency relative to total lung cells is higher in the B cell-deficient mice (S1B and S1D Fig). Together, these data support the notion that there are more cells infiltrating the lungs of *M*. *tuberculosis*-infected WT mice relative to the tuberculous B cell-deficient animals during the chronic phase of infection. These latter data are therefore in line with the observation that B cell-deficiency in mice, in contradistinction to a B cell-sufficient state, is associated with an attenuated inflammatory response in chronic TB. It will be of interest to characterize beyond the CD4^+^ T cell population the composition of lung immune cells in the WT and B cell-deficient mice during chronic tuberculous infection. The results generated should shed light on the effects of B cells on the nature of the granulomatous inflammatory response in chronic TB.

### IL-10R blockade reverses the diminished CD4^+^ T cell phenotype observed in the lungs of B cell-depleted C57BL/6 mice during chronic *M*. *tuberculosis* infection

**Fig 6:**
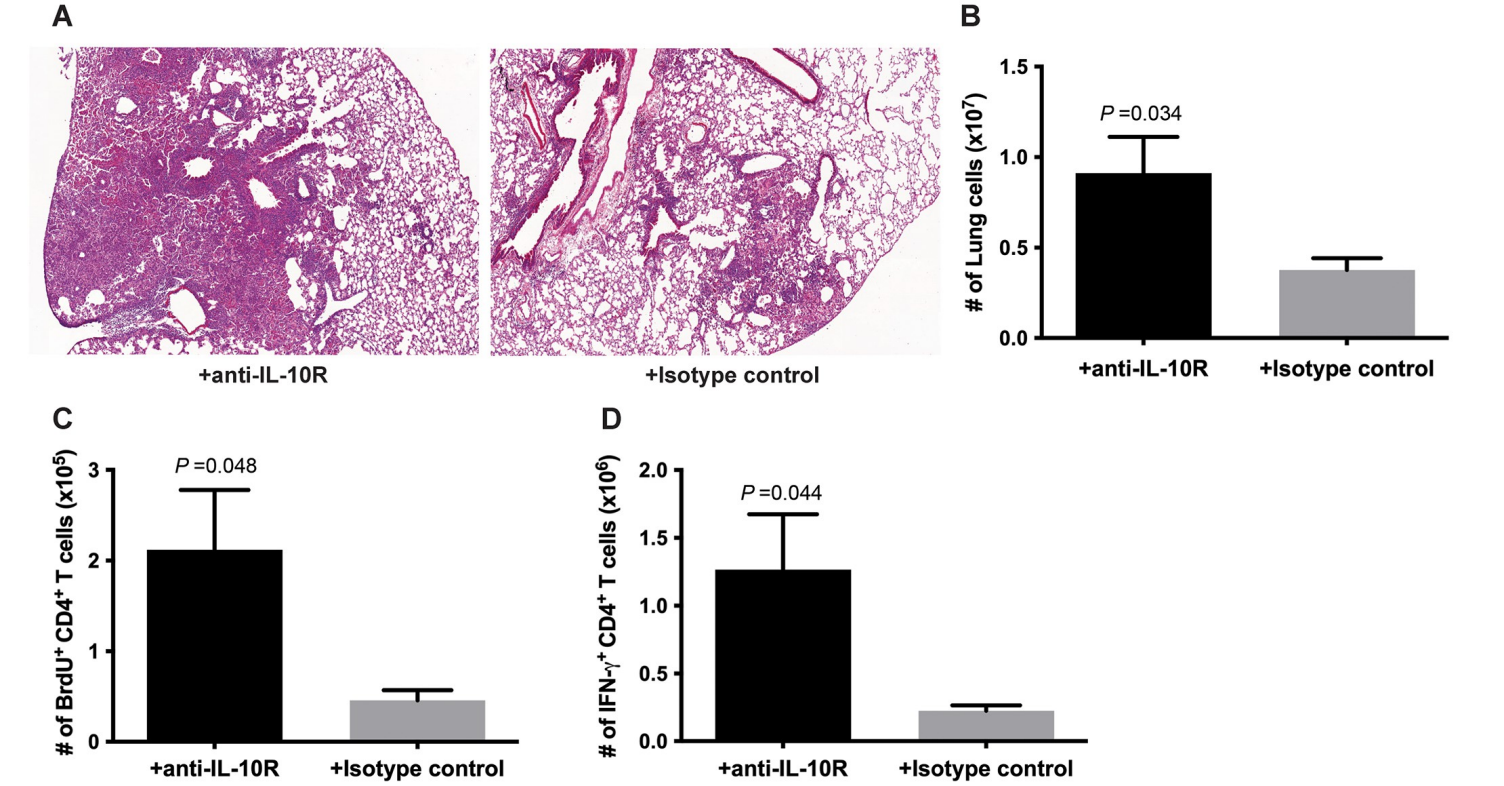
IL-10R blockade reverses the inflammation and diminished CD4^+^ T cell response phenotypes observed in the lungs of B cell-depleted C57BL/6 mice during chronic *M*. *tuberculosis* infection. C57BL/6 mice were depleted for B cells via administration of 5D2 beginning 2 days prior to infection with a low dose (100 CFU) of *M*. *tuberculosis* Erdman delivered by aerosol. B cell depletion was maintained for the duration of the experiment. At 3 months after the infection, IL-10R blockade was initiated using the anti-mouse IL-10R antibody clone 1B1.3A. The control group received non-specific rat IgG. The IL-10R blockade was continued for two months. At five months post-infection (2 months after initiation of IL-10R blockade), mice were sacrificed and analyzed for the levels of inflammation in the lungs, as assessed by histological examination (A) and enumeration of total lung cells (B), CD4^+^ T cells proliferation via BrdU labeling (C), and Th1 response (D). Data shown are representation of two experiments. Three to four mice were analyzed per group. Data depicted in (B), (C), and (D) denote mean ± SEM.

An immunosuppressive property of IL-10 is its capacity to blunt the Th1 response via a variety of mechanisms, such as by modulating macrophage and dendritic cell functions, as well as the migration of Th1 cells [39, 40]. To investigate the contribution of the enhanced IL-10 expression observed in the lungs of B cell-deficient mice to the attenuated levels of pulmonic granulomatous inflammation, as well as to the diminished CD4^+^ T cell proliferation and Th1 immunity, IL-10R blockade experiments were carried out. The monoclonal antibody 1B1.3A (IgG1), which has been shown to effectively block IL-10R signaling in murine experimental TB models, was used for the studies [54–56]. Rat IgG1 served as control antibodies. Histological studies of tuberculous tissue sections revealed that IL-10R blockade during the chronic phase of infection reverses the phenotype of decreased inflammation in the lungs of tuberculous B cell-depleted C57BL/6 mice, as assessed by histological studies (Fig 6A). The total numbers of cells in the lungs of C57BL/6 treated and not treated with IL-10R blockade are 0.91 ± 0.20 x 10^7^ and 0.37 ± 0.07 x 10^7^; respectively (*p*<0.04) (Fig 6B). Further, *in vivo* BrdU labeling showed that B cell-depleted mice receiving 1B1.3A exhibit increased CD4^+^ T cell proliferation: the numbers of BrdU^+^ CD4^+^ T cells of animals treated and untreated with IL-10R blockade are 2.12 ± 0.66 x 10^5^ and 0.46 ± 0.11 x 10^5^; respectively (*p<*0.05) (Fig 6C). The C57BL/6’s blocked for IL-10R signaling also display a significantly enhanced number of Th1 cells (treated mice: 1.27 ± 0.41 x 10^6^ cells; untreated mice: 0.22 ± 0.04 x 10^6^ cells; *p<*0.05) ((Fig 6D). Collectively, results of the IL-10R blockade study showed that IL-10 plays a role in attenuating the level of lung inflammation, as well as in decreasing CD4^+^ T cell proliferation and the Th1 response in B cell-deficient mice during the chronic phase of infection.

### Conspicuous B cell aggregates are present at the periphery of cavitary and necrotic lesions in human tuberculous lungs

**Fig 7:**
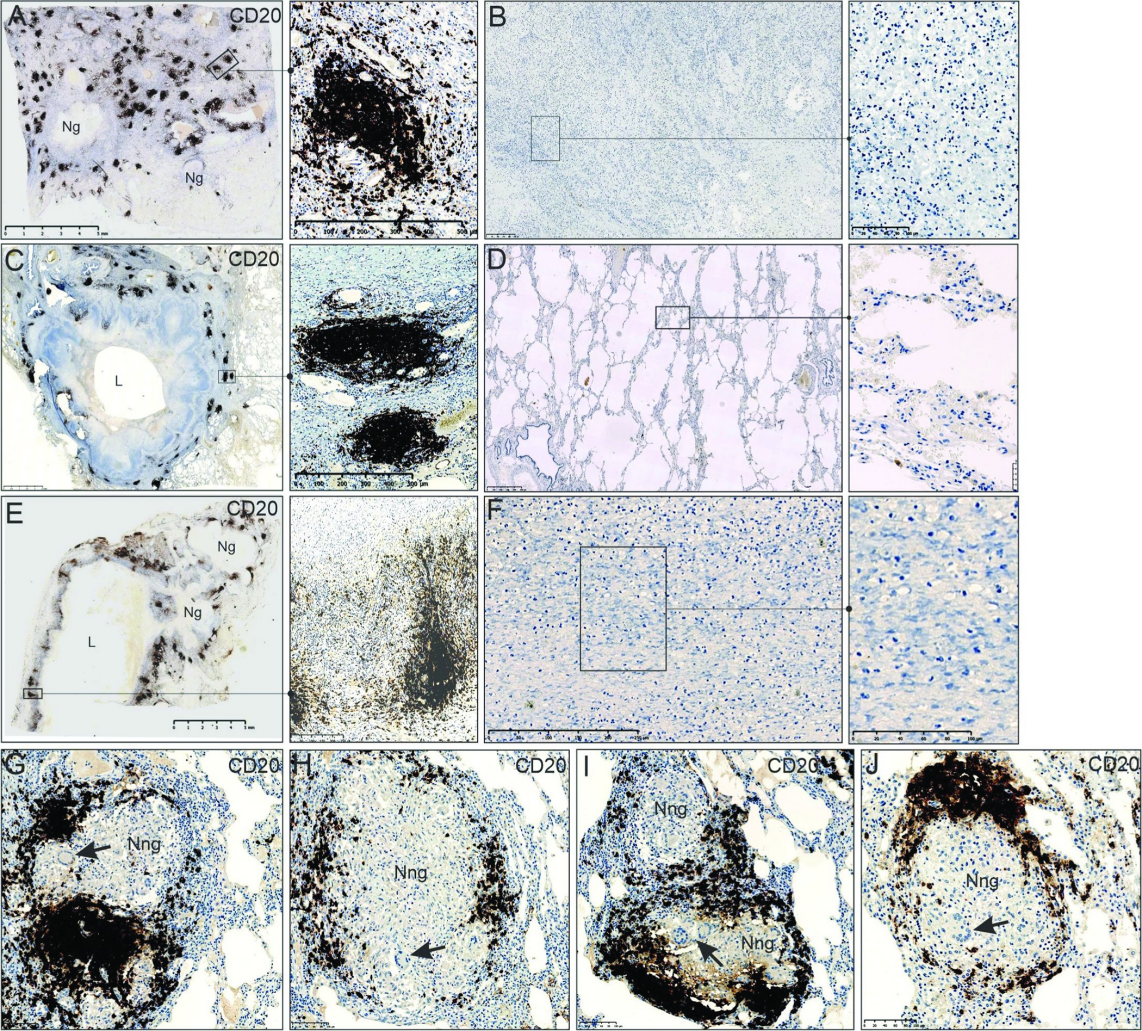
Conspicuous B cell aggregates are present at the periphery of cavitary and necrotic lesions in human tuberculous lungs. (A), (C), and (E) depict low power images demonstrating the histopathology of lung tissue from three patients infected with *M*. *tuberculosis* (see Table 1 for patient information). The medium and high-power images to the right of (A), (C), and (E) illustrate the corresponding B cell aggregates, as detected by anti-CD20 staining. The medium- and high-power images (B), (D), and (F) illustrate regions of tuberculous lung tissue from three patients with varied degree of consolidation (from non-consolidated to markedly consolidated areas) distal to cavitary or necrotic lesions, in which B cells aggregates are conspicuously absent. Photomicrographs of (G-J) show non-necrotic granulomas surrounded by CD20^+^ B cell aggregates, which may represent evolving lesions in transition to a necrotic and/or cavitary state as tissue-damage progresses. Tissue sections were prepared from formalin-fixed, paraffin-embedded lung specimens surgically removed from tuberculous patients. Ng: necrotic granuloma; Nng: non-necrotic granuloma; L: lumen of cavity. Arrows–Langhans giant cells.

The results of the present study have provided evidence that B cells in tuberculous WT C57BL/6 mice with chronic TB can promote lung granulomatous inflammation (Fig 1) in a CD4^+^ T cell-dependent (Figs 2, 3 and 5) and IL-10-dependent manner (Fig 6). However, a role for B cells in promoting inflammation in human tuberculous lungs has not yet been examined. As in the mouse, B cell aggregates, generally thought to be an integral part of ectopic germinal centers capable of modulating local immune response in inflammatory tissues [26,27], are present in tuberculous human lungs [23–25]. Intriguingly, histological examination of surgically procured human tuberculous lung tissues (Table 1) revealed that conspicuous B cell aggregates are located in close proximity to areas of necrosis and cavitation, circumscribing the periphery of the lesions (Fig 7A, 7C and 7E). In contrast, B cells are scanty in areas with no necrosis or cavitation, regardless of the degree of infiltration–moderate (Fig 7D) or abundant (Fig 7B and 7F). There are, however, areas with no evidence of tissue-damaging pathological processes that are associated with conspicuous B cell aggregates (Fig 7G, 7H, 7I and 7J). Although not yet formally investigated, these patterns of topological relation between the B cell aggregates and the necrotic and cavitary lesions raise the possibility that the inflammation-promoting attributes of B cells in chronic murine TB described here may contribute to development of tissue-damaging pathology in the lungs of humans with chronic tuberculous infection. This could occur by the capacity of B cells to modulate the local immune environment. The results suggest the possibility that the progression of immunopathology in the tuberculous lungs, as it pertains to the participation of B cells, begins at an early stage of the granulomatous inflammatory reaction with varied degree of infiltration that is scarcely populated by B cells (Fig 7B, 7D and 7F). As the process progresses, B cells aggregates form at the periphery of the granulomatous lesion (Fig 7G, 7H, 7I and 7J), culminating in subsequent necrotization and cavitation (Fig 7A, 7C and 7E). In sum, the role of pro-inflammatory attributes of B cells in modulating the development of tissue necrosis and cavitation in humans warrants further examination; functional analysis of B cells associated with distinct stages of development of the granulomatous response should be informative in this respect.

**Table 1:** Clinical characteristics of human subjects.

#	Patient #	Age	Sex	Macroscopic and Microscopic features	Type of resection
1	221114LT	30	F	Lung was shrunken, fibrotic and cavitated, weighing 229 g. Lung sections demonstrate fibrocaseous necrosis, interstitial fibrosis, fibro-calcified nodules, prominent lymphocytic infiltrate. Acid fast bacilli were identified, some were present within macrophages.	Left pneumonectomy
2	220314	67	M	Pleural surface of the lobe (323 g) was hemorrhagic, patchy areas of caseous necrosis were observed. Features are consistent with necrotizing granulomatous inflammation; acid-fast bacilli were identified.	Left upper lobectomy
3	221302HW	24	M	Lobectomy specimen weighing 96.9 g showed hemorrhage and cavitated caseous nodule (2 cm in diameter). Areas of necrotizing and non-necrotizing granulomatous inflammation were observed, some granulomas contained suppurative centers, acid-fast bacilli were identified.	Left upper lobectomy
4	224620	38	F	Left lung weighing 281 g showed bronchiectasis and caseating necrosis, which are more pronounced in the upper than the lower lobe. A cavity (2 cm in diameter) was noted in the upper lobe. Acid-fast bacilli were identified; hilar lymph nodes also contain granulomas, but acid-fast bacilli were not demonstrated.	Left pneumonectomy

## Discussion

The present study has provided evidence that B cells can drive the development of lung inflammation in the chronic phase of an *M*. *tuberculosis* Erdman infection (Figs 1 and 5). This enhanced inflammation can, at least in part, be attributed to an augmented CD4^+^ T cell response characterized by increased CD4^+^ T cell proliferation and enhanced levels of IFN-γ-producing CD4^+^ T cells in the lungs of WT mice with chronic TB, relative to the B cell-deficient strains (Figs 2, 3 and 5). IFN-γ can promote inflammation during an immunological reaction [36], including that elicited by respiratory pathogen [57]. Higher levels of IFN-γ expression correlate with worsened immunopathology in *M*. *bovis* infection [58], and granuloma necrosis does not develop in the absence of IFN-γ or its receptor in mice infected with *M*. *avium* [59]. As well, evidence exists that the Th1 cell populations developed in mice upon *M*. *tuberculosis* infection are heterogeneous and specific subsets display pro-inflammatory properties that could be damaging [60–63]. Thus, the decrease in IFN-γ expression in the lungs of B cell-deficient mouse strains may lead to the diminished levels of inflammation observed in these mice (Figs 1 and 5).

IFN-γ is vital to mycobacterial containment in humans [48,64–66] and in mice during both acute [50,51] and chronic [52] infection. Interestingly, despite the significance of IFN-γ in TB control, the diminished expression of IFN-γ-producing CD4^+^ T cells in the lungs of B cell-deficient μMT (Fig 3C) with chronic tuberculous infection, appears not to compromise the restriction of *M*. *tuberculosis* growth, as assessed by quantification of bacterial burden (Fig 1E). It is possible that yet-to-be-defined anti-TB mechanisms are operative in chronic TB to compensate for the attenuated CD4^+^ T cell response observed in tuberculous B cell-deficient mice. Additionally, there is evidence that the IFN-γ response in C57BL/6 mice is expressed at a level in excess of that required for the control of *M*. *tuberculosis* [61]. Thus, the decrease in IFN-γ-producing lung CD4^+^ T cells in B cell-deficient mice during chronic infection might not have diminished below the threshold required for adequate control of *M*. *tuberculosis* growth. The observation raises the possibility that B cells can be targeted to restrict lung inflammation without adversely affecting the control of *M*. *tuberculosis*.

The current study has also revealed that in the lungs of B cell-deficient mice with chronic TB, the expression of IL-10 [37,38] is enhanced relative to WT C57BL/6’s (Fig 4). The anti-inflammatory properties of IL-10 has been shown to limit the development of tissue pathology in a range of infectious diseases [67,68]. In addition, the immunosuppressive property of IL-10 can modulate host susceptibility to a wide variety of microbial pathogens [69–71]. The immunoregulatory IL-10 can suppress dendritic cell and macrophage antigen-presenting capacity, and the migration of Th1 cells to the lungs from the draining lymph nodes [39,40,72], which can lead to attenuation of T cell expansion and development. This latter notion is in line with the results of the *in vivo* BrdU labeling experiment, which show that during the chronic phase of infection, the level of CD4^+^ T cell expansion in C57BL/6’s is more robust than that in B cell-deficient mice; concomitant with lower lung IL-10 expression in the C57BL/6 mice compared to the B cell-deficient group. In addition, B cells are potent antigen presenting cells [23,42–45], which raises the possibility that B cells can directly present antigens to and expand CD4^+^ T cells to promote inflammation. The biological significance of the antigen presenting capacity of B cells to modulate inflammation and influence disease outcome has been demonstrated in a murine multiple sclerosis model [73]. Thus, B cells can indirectly affect the antigen presenting capacity of dendritic cells and macrophages by modulating IL-10 expression, and/or directly present antigens to CD4^+^ T cells to regulate their expansion and development. The two mechanisms are not mutually exclusive. Indeed, evidence exists that the immunosuppressive and anti-inflammatory properties of IL-10 are at play during *M*. *tuberculosis* infection, acting both as a suppressor of immunopathologic progression and as a means by which *M*. *tuberculosis* subverts immunity, thus balancing the development of protective immune response and immunopathology [39,40]. These observations, together with our findings that B cell-deficient mice with chronic TB exhibit diminished lung inflammation that is associated enhanced pulmonic IL-10 expression; and that IL-10 signaling blockade can reverse the inflammation and CD4^+^ T cell phenotypes, suggests that B cells play a role in restricting the expression of the immunosuppressive and anti-inflammatory IL-10 so as to optimize the expression of IFN-γ-producing CD4^+^ T, perhaps at the expense of the risk of the development of host tissue-damaging immunopathology

The levels of a specific cytokine in the locale of an immunological reaction can be modulated by its production and its consumption by immune cells [74]. This consumption is initiated via binding of the cytokine to its receptor expressed on immune cells [74]. The fact that B cells express the IL-10 receptor [40,75], and can be functionally regulated by IL-10 [38] suggests that IL-10 can bind to its receptor on B cells. Thus, the consumption theory can explain, at least in part, the augmented IL-10 levels detected in the lungs of B cell-deficient mice in chronic TB (Fig 4). The multi-functional B cells can also likely regulate the recruitment into the tuberculous lungs of a range of IL-10-producing immune cells [76], thereby affecting lung levels of this cytokine. The expression of IL-10 can be regulated by type I IFN [77,78]. It recently has been reported that the immunosuppressive effects of type I IFN in tuberculous infection can be mediated by IL-10-dependent mechanisms [77–79]. Mycobacterial components can trigger B cells to generate type I IFN, and this cytokine can promote the development of IL-10-producing anti-inflammatory macrophages [79]. The study thus links B cells to type I IFN and IL-10 [79], and possibly inflammation. Of note, although *in vivo* studies involving bone marrow chimeric mice that hyper-express type I IFN exhibit lung inflammation at levels that trend lower than that detected in WT mice during tuberculous infection, the difference does not reach statistical significance [79]. Whether the B cell-type I IFN-IL-10 axis regulates the pulmonic inflammatory response during *M*. *tuberculosis* infection in mice requires additional studies. Finally, it has been reported that B cell-specific IL-4 receptor (IL-4R)-deficient mice exhibit relative resistance to the tubercle bacilli, as assessed by tissue bacterial loads that is associated with diminished tissue inflammation [80]. Thus, whether the attenuated inflammation observed in the lungs of the tuberculous IL-4R-deficient mice compared to WT is directly due to IL-4 signaling through IL-4 responsive B cells, or secondary to the decreased bacillary burden remains to be determined. The mechanisms by which B cells regulate lung inflammation in TB is understudied and are likely complex. Understanding such mechanisms may illuminate how the tubercle bacillus triggers the development of tissue-damaging immunopathology.

In the chronic *M*. *tuberculosis* Erdman TB model used in the present study, the diminished IFN-γ-producing CD4^+^ T cell response and augmented IL-10 expression in the lungs of B cell-deficient mice does not adversely affect the capacity of the infected host to control *M*. *tuberculosis* (Fig 1E), even though IFN-γ plays an important in host defense against the tubercle bacillus [48,50–52,64–66] and IL-10 can compromise anti-TB immunity [39,40]. In fact, there is a substantial difference in the median survival time between the C57BL/6 and μMT mice in the 100 CFU Erdman model in a period (~8 months to 11 months post-infection) in the chronic phase of infection, during which WT’s succumb to the infection earlier and at a more rapid kinetics than B cell-deficient mice (Fig 1F). This survival phenotype is apparent in three independent experiments. The results suggest that there is a period during the chronic phase of TB when the B cell-driven enhanced inflammatory response can be detrimental to the host. It is possible that as disease exacerbation sets in during the terminal stage of the infection, tuberculous mice succumb to the infection regardless of the B cell status. Nevertheless, the data suggest that there is a period in the chronic phase of infection during which manipulation of B cells might result in better disease outcome.

The observation that B cells drive the level of granulomatous inflammation in the lungs of B cell-sufficient mice with chronic Erdman TB is congruent with the results of a previous report that showed decreased pulmonary infiltrates in μMT mice chronically infected with the clinical isolate CDC1551 of *M*. *tuberculosis* [33]. These results suggest the inflammation-regulatory function of B cells in chronic TB is independent of the causative mycobacterial strains. The validity and generality of this notion remains to be tested with additional clinical *M*. *tuberculosis* strains. Worthy of note, while the pro-inflammatory attribute of B cells is apparent during both acute and chronic infection established by CDC1551, this property of B cells is observed only in the chronic phase of the Erdman infection. In the Erdman model, B cells display an anti-inflammatory phenotype during the acute phase of infection [31,35]. That B cells regulate inflammation in diametrically opposed manner during the acute and chronic phase of the Erdman infection (acute infection–anti-inflammatory; chronic infection–pro-inflammatory) suggest the roles of B cells in modulating the host response to *M*. *tuberculosis* can be infection phase-dependent. This discrepancy could be driven by specific sets of *M*. *tuberculosis* antigens differentially expressed in mice during acute versus chronic Erdman TB, thus rendering the local lung immunological environments in the two phases of infection distinct. Indeed, ample evidence exist that *M*. *tuberculosis* can adapt to environmental signals during infection by modulating its cell envelope constituents, including moieties that are immunologically active [81–85].

The results showing that in acute TB, B cells in mice infected with the Erdman and CDC1551 isolates exhibit anti- and pro-inflammatory attributes; respectively, suggests that the inflammation-regulatory properties of B cells in the acute phase of infection can be strain-dependent. This strain-dependency could be secondary to the intrinsic disparity in the expression of specific antigens by the two mycobacterial strains during acute infection. Indeed, the phenotypic differences in the immune response to *M*. *tuberculosis* CDC1551 and the laboratory strain H37Rv have been attributed to the discrepancy in lipid moieties and cell wall components of the two isolates [86]. Together, the distinct patterns of lung inflammation in B cell-deficient mice with TB caused by the clinical isolate CDC1551 and the laboratory Erdman strain suggest the possibility that the role of B cells in regulating inflammation in TB is intricately intertwined with the intrinsic property of the causative isolates as well as the phase of infection.

The relevance and the significance of the intrinsic properties of *M*. *tuberculosis* in differentially regulating the inflammatory response in the lungs of humans with TB and in affecting disease outcome has been underscored by recent studies that involve the analysis of high-transmission and low-transmission clinical isolates. These two categories of clinical isolates studied elicit distinct patterns of inflammation and transmissibility [87,88]. Whether B cells play a role in regulating the granulomatous response in the lung of humans infected with the high-transmission and low-transmission *M*. *tuberculosis* strain remains to be evaluated. Worthy of note, we have observed that in the lungs of humans with chronic TB, conspicuous B cells aggregates are topologically associated with cavitary and necrotic lesions (Fig 7). While the significance of this association remains to be determined, it is tempting to speculate that B cells contribute to the development of immunopathological damage in the lungs of humans during chronic *M*. *tuberculosis* infection. Study of the B cells aggregates from human tuberculous lungs will inform about the roles for B cells in mediating the development of transmission-promoting tissue-damaging inflammation in chronic TB and may lead to the design of strategies that can improve the control of *M*. *tuberculosis*.

In summary, the present study has provided evidence that in chronic TB, B cells play a significant role in driving the lung inflammatory response in an IL-10 and Th1/IFN-γ-dependent manner. The data suggest that B cells can drive directly or indirectly restrict the pulmonic expression of the anti-inflammatory and immunosuppressive IL-10, and as a result, optimizing protection by augmenting the development of the protective and pro-inflammatory Th1 immunity, thereby enhancing inflammation that may cause host-detrimental immunopathology. The reversal of the attenuated inflammatory and Th1 response in the B cell-deficient tuberculous mice by IL-10R blockade support the notion that IL-10 is an important factor in fine tuning the protective, pro-inflammatory IFN-γ/Th1 immune response in chronic TB. The results have also provided evidence that the role of B cells in modulating the inflammatory response in TB can be mycobacterial strain- and infection phase-dependent. This latter observation, together with the multifunctionality of B cells, predict that the mechanisms by which these lymphocytes regulate lung inflammation in chronic TB are complex, and likely involves additional elements beyond the B cell/IL-10/Th1 axis identified by the present study. Finally, the association of B cell aggregates with necrotic and cavitary lesions in the lungs of patients with TB raises the possibility that B cells may contribute to the development of tissue-damaging pathology in humans infected with the tubercle bacillus.

## Materials and methods

### Ethics statement

The human lung pathology study was approved by the University of KwaZulu-Natal Biomedical Research Ethics Committee (BREC, Class approval study number BCA 535/16). Patients undergoing lung resection for TB, their study protocol, associated informed consent documents, and data collection tools were approved by the UKZN BREC (Study ID: BE 019/13). Animal studies were conducted in accordance with the National Institutes of Health guidelines in compliance with assurance of the well-being of laboratory animals. The Institutional Animal Care and Use Committee of Albert Einstein College of Medicine have approved the animal protocols used in this study. The protocol number is 00001304.

### Mice

Female mice, C57BL/6 (Charles River, Rockland, MA) or B-cell deficient (backcrossed to C57BL/6 mice; μMT, Jackson Laboratories, Bar Harbor, ME) [34], 8-to- 10-weeks old, were used in all experiments. All mice were housed in a biosafety-level-3 animal laboratory and maintained pathogen-free by routine serological and histopathological examinations.

### Mycobacteria and mouse infection

Maintenance of mycobacterial stocks and propagation of bacteria, as well as mouse infection were carried out as previously described [31]. Bacterial stock of *M*. *tuberculosis* strain Erdman (Dr. Frank Collins, Trudeau Institute, Saranac Lake, NY) was prepared by passage through mice to maintain virulence, expanded once by culture in 7H9 liquid medium (Difco), and stored in 3x10^8^ bacilli/ml aliquots at -80°C until use. Before infection, aliquot was thawed, diluted 1:10 in PBS with 0.05% Tween 80 (Sigma), and sonicated to achieve uniform suspension. Mice were infected by aerosol using the Lovelace nebulizer (In-Tox Products, Moriarty, NM) with *M*. *tuberculosis* diluted to a concentration calibrated to deliver approximately 100 bacilli to the lungs. The 100 CFU dose is considered the “conventional” inoculum used in the murine tuberculosis model. Inoculum dose was confirmed by colony counts on 7H10 agar plates (Difco) of whole lung homogenates at 16 to 24 hours post-aerosol for each experiment.

### CFU enumeration of infected tissues in mice

Tissue bacterial loads were quantified as previously described [31]. At indicated intervals after infection, tissue bacterial burden was quantified by plating serial dilutions of lung, liver, and spleen homogenates onto 7H10 agar plates. In all experiments, two right lung lobes or approximately one-third of the lung, approximately one-eighth of the liver, and approximately one-half of the spleen were used for enumerations of tissue bacterial burden. Bacterial burden was assessed as CFU, determined by the number of colonies on plates after incubation at 37°C for 21 days.

### Murine histopathological studies

Tissue samples from lungs were fixed in 10% buffered formalin and subsequently embedded in paraffin [31]. Serial 5 to 6 μm sections were stained with hematoxylin and eosin (H&E) and photographed under a light microscope as previously described [31].

### Preparation of single-cell suspension from mouse lung tissues

Single cell suspension from mouse lungs was prepared as previously described [31]. In all experiments, left lungs were aseptically removed, minced using sterile razor blades (Fisher Scientific), and incubated in RPMI 1640 containing 1 mg/ml collagenase (Sigma) and 30 ug/ml DNase (Sigma) at 37°C for 60 min. To achieve a single-cell suspension, lung fragments were pressed through a 70 μm pore nylon cell-strainer (BD Falcon) using the flat-end of a sterile 3 ml syringe plunger. Cells were washed twice in complete RPMI (RPMI supplemented with 2mM L-glutamine, 25mM HEPES, 10% fetal bovine serum and 55 μM 2-mercaptoethanol) and red blood cells were lysed by incubation in ACK lysis solution (Thermofisher Scientific; 0.156 M NH_4_Cl, 10 mM KHCO_3_, 0.1 mM Na_2_EDTA, pH 7.2) for 5 min at room temperature. Cells were then washed again in complete RPMI and counted, using trypan blue to exclude dead cells. Total number of cells from the lungs was enumerated using a hemocytometer in conjunction with light microscopy [31].

### *Ex vivo* lung cell culture and ELISA of tuberculous mice

Cultures of lung cells and quantification of the levels of cytokines in culture supernatants were carried out as described previously [31]. For *ex vivo* cell culture, single-cell suspension prepared from the lung were cultured in complete RPMI alone or with 10 μg/ml mycobacterial protein purified derivative (PPD) (Statens Serum Institute, Copenhagen) at 1.0 x 10^7^ cells per ml. After 48 hours of culture, supernatants were harvested, filter sterilized, and stored at -20°C. Matched antibody pair for IL-10 used for ELISA were purchased from R&D Systems (Minneapolis, MN) and utilized according to manufacturer’s protocol. Where indicated, B-cells were depleted from single-cell lung suspensions from *M*. *tuberculosis*-infected C57BL/6 mice using CD19^+^ MACS beads (Miltenyi Biotec) prior to *ex vivo* culture. This method routinely achieves above 95% B cells depletion, as assessed by flow cytometry analysis. For *ex vivo* carboxyfluorescein succinimidyl ester (CFSE, molecular probes) dilution assay, lung cells were incubated in 10 μM CFSE at 37°C for 10 min before quenching on ice. Cells were subsequently cultured for 5 days at 37°C and CFSE fluorescence was detected by flow cytometry.

### Intracellular cytokine staining and flow cytometric analysis of lungs cells from *M*. *tuberculosis*-infected mice

Flow cytometric analysis of cells stained for intracellular cytokines were conducted as described previously [31]. Monoclonal antibodies against the following antigens were purchased from BD Biosciences (anti-CD4-FITC (clone GK1.5), anti-CD4-PE (clone GK1.5), anti-IFN-γ-PE (clone XMG1.2)) or Thermofisher Scientific (anti-CD3-APC (clone 145-2C11), anti-CD45-efluor450 (clone 30-F11)). Dead cells were excluded by the LIVE/DEAD Fixable blue Dead Cell Stain Kit (Thermofisher Scientific) according to manufacturer’s protocol. For intracellular cytokine staining, lung single-cell suspensions prepared as described above were stimulated in wells of 96 well plates in complete RPMI for 2 h at 37°C and 5% CO_2_ in the presence of PMA (50 ng/ml) and ionomycin (500 ng/ml). Brefeldin A (1 ug/ml; Thermofisher Scientific) was added to wells and incubation was continued for another 2 h. For some experiments, lung cells were stimulated with plate bound anti-CD3 antibody (10 ug/ml; BD Biosciences). Subsequently, a cytofix/cytoperm kit (BD Biosciences) was utilized according to manufacturer’s specifications for intracellular cytokine staining. For *in vivo* bromodeoxyuridine (BrdU) labeling, 1 mg BrdU (BD Biosciences) was injected into mice 16–20 h before sacrificing mice and single lung cells prepared as described above were used for BrdU staining following manufacturer’s protocol of the FITC BrdU Flow Kit (BD Biosciences). Flow cytometry data were acquired on an LSR II (BD Biosciences) and analyzed using FlowJo software v.10 (BD Biosciences).

### B cell depletion in mice

The anti-CD20 monoclonal antibody (mAb; Genentech) 5D2 was used to deplete B cells from WT C57BL/6 mice as described previously [35]. Briefly, mice received a dose of 5D2 (10 mg/kg per mouse) administered intravenously 2 days prior to aerosol infection. Two weeks after the initial treatment, the 5D2 mAb was administered intraperitoneally at an every-other-week dosing regimen to maintain effective B cell depletion until analysis at 20 weeks after a low-dose aerosol infection (100 CFU) of *M*. *tuberculosis* Erdman. Control mice received injection with phosphate-buffered saline (PBS).

### IL-10 blockade study in tuberculous mice

C57BL/6 mice were infected by aerosol with 100 CFU of *M*. *tuberculosis* Erdman. At day 90 post infection, 1 mg of anti-mouse IL-10 receptor (IL-10R; clone 1B1.3A; BioXcell) or isotype control Rat IgG1 antibody (BioXcell) was administered intraperitoneally (i.p.) per mouse. One week later, IL-10R was delivered at a dose of 0.2 mg i.p. once a week for two months, at which point mice were sacrificed and analyzed for the levels of inflammation in the lungs (assessed by histological examination and enumeration of total lung cells), CD4^+^ T cells proliferation via BrdU labeling, and Th1 response (Elispot) as described above. The regimen of IL-10R Ab administration derived from published results pertaining to murine experimental TB models with modification [54–56].

### Human lung study

The human study was approved as stated in the ethics statement. The samples used in the present studies were obtained from HIV-negative individuals. These human samples were procured from the period 2009–2010, and therefore were from the time before the onset of the COVID-19 pandemic. *M*. *tuberculosis*-infected human lung tissues are routinely obtained following surgery for removal of irreversibly damaged lobes or lungs (bronchiectasis and/or cavitary lung disease). Patients were assessed for extent of pulmonary disease (cavitation and or bronchiectasis) via HRCT. The fitness of each patient to withstand a thoracotomy and lung resection was determined by Karnofsky score, six-minute walk test, spirometry, and arterial blood gas. Assessment of patients with massive hemoptysis included their general condition, effort tolerance prior to hemoptysis, arterial blood gas measurement, serum albumin level and HRCT imaging of the chest. On gross assessment, all pneumonectomies or lobectomies were bronchiectatic, hemorrhagic, variably fibrotic and atelectatic and contained visible tubercles (Table 1). Written informed consent was obtained from patients recruited from King DinuZulu Hospital Complex, a tertiary center for TB patients in Durban, South Africa. Detailed methods for histopathological studies, including histology slide digitization and protocols for immunohistochemistry are presented in S1 Text.

### Statistical analyses

Statistical significance was assessed by the unpaired Student’s *t* test (GraphPad Prism 9.4.1 software) as previously described [31,35]. *p* values < 0.05 were considered statistically significant.

## Supporting information

S1 Fig The CD4^+^ T cell population in the lungs of B cell-deficient mice during chronic Mtb infection. WT C57BL/6 and B cell-deficient (μMT and anti-CD20 mAb-treated WT) mice were aerogenically infected with 100 CFU Mtb Erdman. At five months post-infection, lung cells were procured and subjected to flow cytometric analysis. The data denote the absolute number of CD4^+^ T cells in the lungs (A and C), and the frequency of CD4^+^ T cells relative to total lung cells (B and D). The data shown were derived from the experiments depicted in Figs 2 and 3 (the μMT study, which corresponds to S1A and S1B Fig), and Fig 5 (the anti-CD20 study, which corresponds to S1C and S1D Fig) in the manuscript. Three to five animals per group were studied. * p<0.05; ** p<0.005; **** p< 0.0001.
(TIF)

S1 Text Supplementary Materials and Methods. (DOCX)
