# Supplementary figures and images for: B cells promote granulomatous inflammation during chronic Mycobacterium tuberculosis infection in mice
Source: PLoS Pathog. 2023 Mar 8;19(3):e1011187. doi: 10.1371/journal.ppat.1011187 (PMC9994760; doi:10.1371/journal.ppat.1011187)

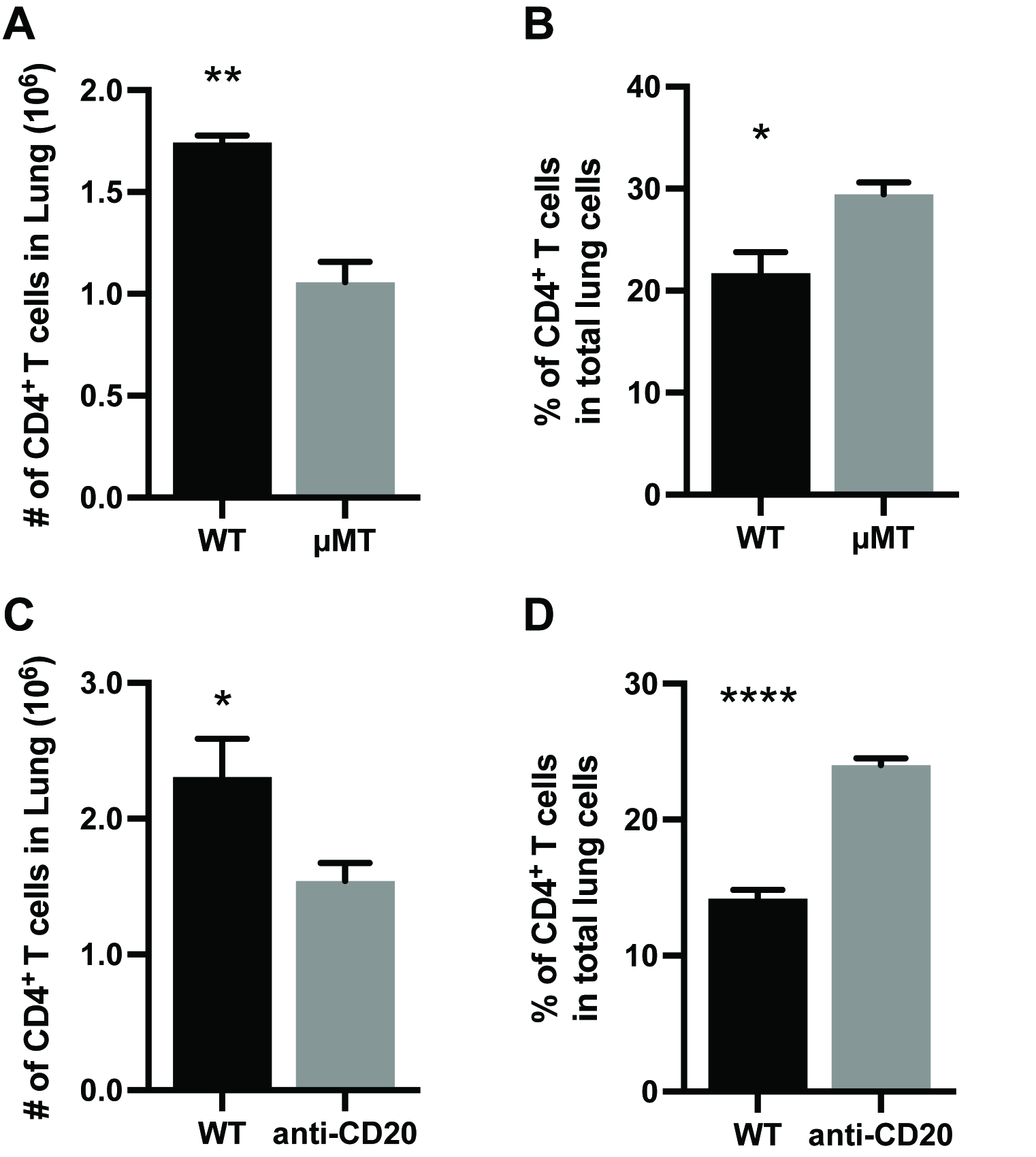

Supplement: S1 Fig — WT C57BL/6 and B cell-deficient (μMT and anti-CD20 mAb-treated WT) mice were aerogenically infected with 100 CFU Mtb Erdman. At five months post-infection, lung cells were procured and subjected to flow cytometric analysis. The data denote the absolute number of CD4+ T cells in the lungs (A and C), and the frequency of CD4+ T cells relative to total lung cells (B and D). The data shown were derived from the experiments depicted in Figs 2 and 3 (the μMT study, which corresponds to S1A and S1B Fig), and Fig 5 (the anti-CD20 study, which corresponds to S1C and S1D Fig) in the manuscript. Three to five animals per group were studied. * p<0.05; ** p<0.005; **** p< 0.0001. (TIF) [file ppat.1011187.s001.tif]
