# Supplementary material for: B cells promote granulomatous inflammation during chronic Mycobacterium tuberculosis infection in mice
Source: PLoS Pathog. 2023 Mar 8;19(3):e1011187. doi: 10.1371/journal.ppat.1011187 (PMC9994760; doi:10.1371/journal.ppat.1011187)
Supplement: S1 Text — (DOCX) [file ppat.1011187.s002.docx]

**Supporting information**

**S1Text. Supplementary Materials and Methods**

**Histopathology**

Identification of anatomical features and pathology was confirmed by histological techniques using Hematoxylin and Eosin (H&E) stain. Briefly, samples of lung were aseptically removed and fixed in 10% buffered formalin and processed in a vacuum filtration processor using a xylene-free method with isopropanol as the main substitute fixative. Tissue sections were embedded in paraffin wax. Sections were cut at 4 µm, baked at 60°C for 15 min, dewaxed through two changes of xylene and rehydrated through descending grades of alcohol to water. These sections were stained with H&E or the Masson’s trichrome method using standard procedures. Slides were dehydrated in ascending grades of alcohol, cleared in xylene, and mounted with a mixture of distyrene, plasticizer, and xylene.

**Histology slide digitization**

Human lung specimens were digitized using a Hamamatsu NDP slide scanner (Hamamatsu NanoZoomer RS2, Model C10730-12) and its viewing software (NDP.View2). The red, green, and blue color balance was kept at 100% whereas gamma correction was maintained between 0.7 and 2. Brightness (60–110%) and contrast (100–180%) settings vary slightly between slides depending on staining quality. Resolution was 230 nm/pixel yielding file sizes of 2-4.4 GB. Contrast, brightness, and intensity of exported images (jpg format) were minimally adjusted using CorelDraw 2020.

**Immunohistochemistry**

Pulmonary tissue was cut into 2-4 µm thick sections, mounted on charged slides, and heated at 56° C for 15 min. Sections were dewaxed in xylene followed by rinse in 100% ethanol and one change of SVR (95%). Slides were then washed under running water for two min followed by antigen retrieval via Heat Induced Epitope Retrieval (HIER) in Tris-sodium chloride (pH 6.0) for 30 min. Slides were cooled for 15 min and rinsed under running water for two min. Endogenous peroxide activity was blocked using 3% hydrogen peroxide for 10 min at room temperature (RT). Slides were then washed in PBST and blocked with protein block (Novolink) for 5 min at room temperature. Sections were incubated with primary antibodies for CD20 (M0755-CD20cy-L26, DAKO, 1:1000), followed by washing and incubation with the polymer (Novolink) for 30 min at RT. Slides were then washed and stained with DAB for 5 min, washed under running water and counterstained with hematoxylin for 2 min. Slides were rinsed under running water, blued in 3 % ammoniated water for 30s, washed under water, dehydrated, and mounted in Distyrene Plasticizer Xylene (DPX). For isotype control sections, a similar protocol to our previous studies was followed [1,2]; either IgG4 (LS-C70325/27332) or rabbit IgG (ab37415, m) was used (at the same concentration/dilution as the primary antibodies) in place of the primary antibodies (isotype control).

**References**

1. Chinta KC, Rahman MA, Saini V, Glasgow JN, Reddy VP, et al. (2018) Microanatomic Distribution of Myeloid Heme Oxygenase-1 Protects against Free Radical-Mediated Immunopathology in Human Tuberculosis. Cell Rep 25: 1938-1952 e1935. [https://doi.org/10.1016/j.celrep.2018.10.073 PMID: 30428359](https://doi.org/10.1016/j.celrep.2018.10.073%20PMID:%2030428359)

2. Reddy VP, Chinta KC, Saini V, Glasgow JN, Hull TD, et al. (2018) Ferritin H Deficiency in Myeloid Compartments Dysregulates Host Energy Metabolism and Increases Susceptibility to Mycobacterium tuberculosis Infection. Front Immunol 9: 860. [https://doi.org/10.3389/fimmu.2018.00860 PMID: 29774023](https://doi.org/10.3389/fimmu.2018.00860%20PMID:%2029774023)
